# Supplementary material for: The interactome of intact mitochondria by cross-linking mass spectrometry provides evidence for coexisting respiratory supercomplexes
Source: Mol Cell Proteomics. 2017 Dec 8;17(2):216–32. doi: 10.1074/mcp.RA117.000470 (PMC5795388; doi:10.1074/mcp.RA117.000470)
Supplement: Supplemental Data [file supp_RA117.000470_133922_0_supp_23978_fzffwf.zip › spectra_annotation/mito_spectra_annotation/341-1-8-1-8-1.pdf]

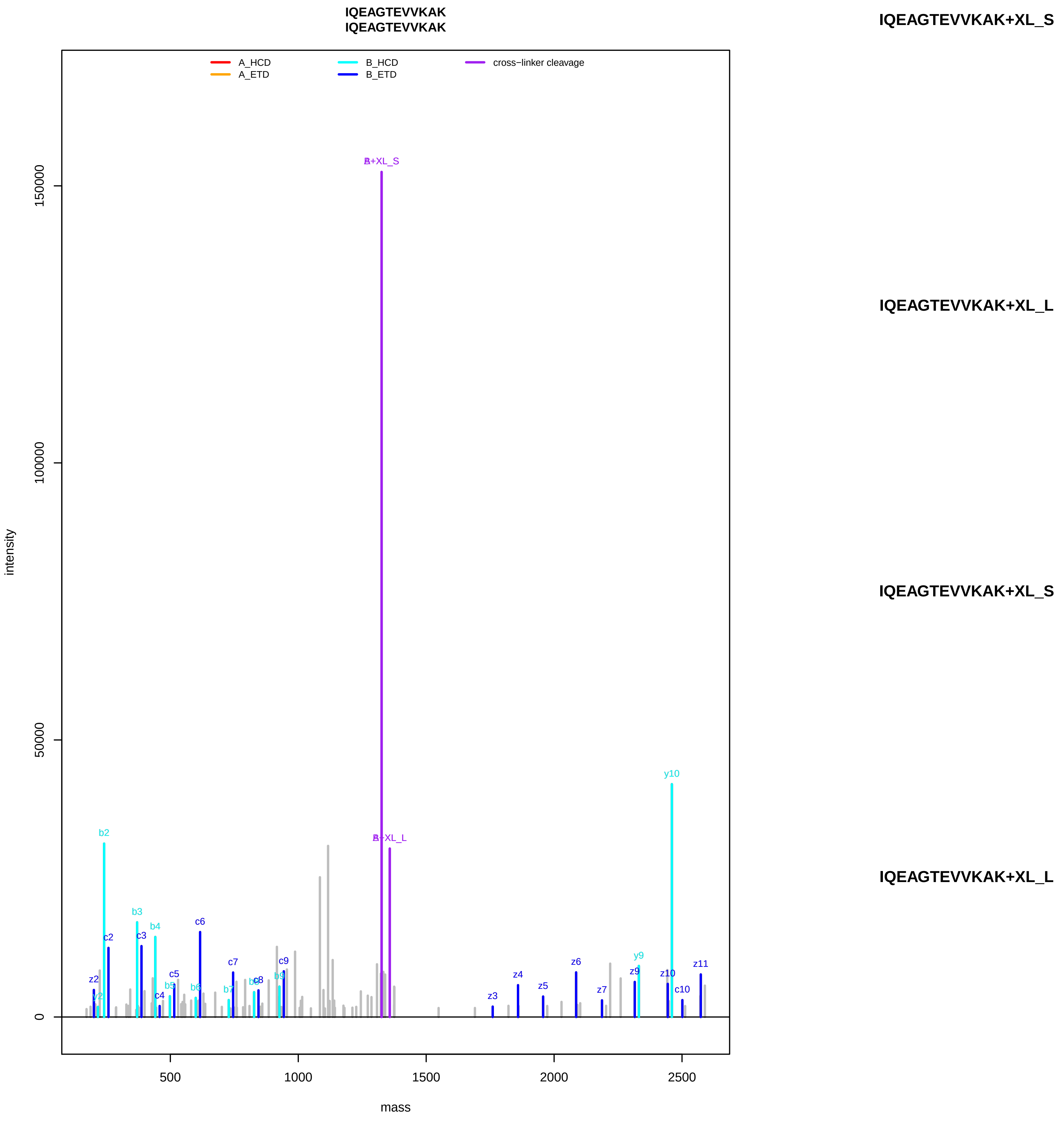
IQEAGTEVVKAK
IQEAGTEVVKAK
A_HCD
A_ETD
B_HCD
B_ETD
cross−linker cleavage
intensity
mass
0
50000
100000
150000
500
1000
1500
2000
2500
A+XL_S
B+XL_S
A+XL_L
B+XL_L
b2
b3
b4
b5
b6
b7
b8
b9
y2
y9
y10
c2
c3
c4
c5
c6
c7
c8
c9
c10
z2
z3
z4
z5
z6
z7
z9
z10
z11
IQEAGTEVVKAK+XL_S
IQEAGTEVVKAK+XL_L
IQEAGTEVVKAK+XL_S
IQEAGTEVVKAK+XL_L
